# Cannabis Use Disorder Emergency Department Visits and Hospitalizations and 5-Year Mortality

**DOI:** 10.1001/jamanetworkopen.2024.57852

**Published:** 2025-02-06

**Authors:** Daniel T. Myran, Michael Pugliese, André J. McDonald, Jennifer Xiao, Benedikt Fischer, Yaron Finkelstein, Peter Tanuseputro, Joseph Firth, Amir Pakpour, Chih-Wei Hsu, Wing-Chung Chang, Marco Solmi

**Affiliations:** 1 Department of Family Medicine, University of Ottawa, Ottawa, Ontario, Canada; 2 Ottawa Hospital Research Institute, Ottawa, Ontario, Canada; 3 ICES uOttawa, Ottawa Hospital Research Institute, Ottawa, Ontario, Canada; 4 Bruyère Research Institute, Ottawa, Ontario, Canada; 5 School of Epidemiology and Public Health, Faculty of Medicine, University of Ottawa, Ottawa, Ontario, Canada; 6 Peter Boris Centre for Addictions Research, St Joseph’s Healthcare Hamilton, Hamilton, Ontario, Canada; 7 Michael G. DeGroote Centre for Medicinal Cannabis Research, McMaster University, Hamilton, Ontario, Canada; 8 Faculty of Health Sciences, Simon Fraser University, Vancouver, British Columbia, Canada; 9 Research & Graduate Studies, University of the Fraser Valley, Abbotsford, British Columbia, Canada; 10 Department of Psychiatry, University of Toronto, Toronto, Ontario, Canada; 11 Department of Psychiatry, Federal University of Sao Paulo, São Paulo, Brazil; 12 School of Population Health, University of Auckland, Auckland, New Zealand; 13 Divisions of Emergency Medicine and Clinical Pharmacology and Toxicology, Department of Paediatrics, The Hospital for Sick Children, Toronto, Ontario, Canada; 14 Department of Pediatrics, University of Toronto, Toronto, Ontario, Canada; 15 Department of Pharmacology and Toxicology, University of Toronto, Toronto, Ontario, Canada; 16 Division of Psychology and Mental Health, University of Manchester, Manchester Academic Health Science Centre, Manchester, United Kingdom; 17 Social Determinants of Health Research Center, Research Institute for Prevention of Non-Communicable Diseases, Qazvin University of Medical Sciences, Qazvin, Iran; 18 Department of Nursing, School of Health and Welfare, Jönköping University, Jönköping, Sweden; 19 Department of Psychiatry, Kaohsiung Chang Gung Memorial Hospital and Chang Gung University College of Medicine, Kaohsiung, Taiwan; 20 Department of Psychiatry, School of Clinical Medicine, Li Ka Shing Faculty of Medicine, The University of Hong Kong, Hong Kong SAR, China; 21 State Key Laboratory of Brain & Cognitive Sciences, The University of Hong Kong, Hong Kong SAR, China; 22 Department of Psychiatry, University of Ottawa, Ottawa, Ontario, Canada; 23 Department of Mental Health, The Ottawa Hospital, Ottawa, Ontario, Canada; 24 Department of Child and Adolescent Psychiatry, Charité Universitätsmedizin, Berlin, Germany

## Abstract

**Importance:**

Cannabis use disorders (CUD) are associated with adverse health effects, including mental disorders and motor vehicle collision-related injuries. However, little is known about whether CUDs are associated with increased mortality risk.

**Objective:**

To examine whether individuals receiving incident hospital-based care (an emergency department visit or hospitalization) for a CUD is associated with increased risk of death.

**Design, Setting, and Participants:**

This population-based retrospective cohort study included all individuals aged 15 to 105 years living in Ontario, Canada, between 2006 and 2021 (n = 11 622 571 individuals). Overall and cause-specific mortality were compared between individuals with incident hospital-based CUD care and age- and sex-matched members of the general population or individuals with hospital-based care for other substance use disorders using cause-specific hazard models adjusted for comorbid mental health, substance use, and chronic health conditions. Statistical analysis was performed from September to December 2024.

**Exposure:**

Incident hospital-based CUD care.

**Main Outcomes and Measures:**

Overall and cause-specific mortality identified using vital statistics.

**Results:**

The matched analysis included 527 972 individuals (mean [SD] age, 29.9 [13.6] years; 330 034 [62.5%] male) with a median (IQR) follow-up of 5 (3-9) years; 106 994 had incident CUD. Within 5 years of incident hospital-based CUD care, 3770 individuals (3.5%) died compared with 2550 (0.6%) of matched general population members. After adjusting for comorbid conditions, individuals with incident hospital-based CUD care were at increased risk of death relative to the general population (adjusted hazard ratio [aHR], 2.79 [95% CI, 2.62-2.97]). Individuals with hospital-based CUD care were at increased risk of all investigated types of death and particularly elevated risk of death by suicide (aHR, 9.70 [95% CI, 6.04-15.57]), trauma (aHR, 4.55 [95% CI, 3.55-5.82]), opioid poisoning (aHR, 5.03 [95% CI, 2.86-8.84]), other drug poisonings (aHR, 4.56 [95% CI, 3.11-6.68]), and lung cancer (aHR, 3.81 [95% CI, 2.39-6.07]) relative to the general population. Compared with an individual with hospital-based care for CUD, individuals with hospital-based care for alcohol (aHR, 1.30 [95% CI, 1.26-1.34]), stimulants (aHR, 1.69 [95% CI, 1.62-1.75]), and opioids (aHR, 2.19 [95% CI, 2.10-2.27]) were at relatively increased risk of death within 5 years.

**Conclusions and Relevance:**

In this cohort study of all residents of Ontario, Canada, individuals with incident hospital-based CUD care were at markedly increased risk of death compared with the general population. These findings suggest important clinical and policy implications, given global trends toward cannabis legalization and market commercialization accompanied by increasing cannabis use and CUDs.

## Key Points

**Question:**

Are individuals who have hospital-based (emergency department or hospitalization) care for a cannabis use disorder (CUD) at increased risk of death?

**Findings:**

In this cohort study of 11.6 million people studied for a median of 5 years, individuals with incident hospital-based care for a CUD were at a 2.8-fold increased risk of death within 5 years relative to the general population.

**Meaning:**

These results suggest that individuals who require hospital-based care for a CUD may be at increased risk of premature death.

## Teaser

This cohort study investigates whether incident hospital-based care (an emergency department [ED] visit or hospitalization) for cannabis use disorder is associated with increased risk of death among individuals in Ontario, Canada.

## Introduction

Cannabis use is increasing globally and is the third most commonly used drug after alcohol and nicotine. Cannabis use is associated with the development of several major psychiatric illnesses, with the largest body of evidence being for psychosis and schizophrenia.^1,2,3,4,5^ Prior research has shown associations between cannabis use and increased risk of self-harm and suicide in adolescents and young adults.^6,7^ Intoxication with tetrahydrocannabinol (THC), the psychoactive component of cannabis, is also associated with a greater risk of fatal motor vehicle collisions.^8^ Growing data suggest that cannabis legalization and particularly commercialization of cannabis—allowing widespread retail access and promotion to cannabis—may result in increases in cannabis use, cannabis use disorders (CUDs), and other associated harms.^9,10,11,12^ Importantly, despite large increases over time in patterns of cannabis use that are associated with CUDs, including daily use and high potency use, evidence on the association between CUDs and mortality is limited.^13^

A single study of 6445 individuals treated for CUD in Denmark reported a 4.7 standardized mortality ratio relative to the general population.^13^ However, the study only included 142 deaths in the CUD group, captured a very young cohort, and did not account for comorbid substance use disorders.^13^ CUDs in youths with mood disorders have been found to be associated with increased risk of all-cause mortality.^6^ A larger number of studies have examined the association between differing levels of cannabis use and mortality, with some evidence suggesting that more frequent cannabis use may be associated with increased risk of death. A study of 121 895 participants in the United Kingdom biobank found that self-reported “heavy cannabis use” (>100-lifetime episodes of cannabis use) was associated with a 1.49-fold increase in all-cause mortality and a 2.67-fold increase in cardiovascular mortality in females relative to never users.^14^ Longitudinal studies of Swedish men registering for compulsory military training found that individuals who had self-reported using cannabis more than 50 times by 19 years of age had a modest elevation in mortality risk over 40 years of follow-up.^15,16^ US studies have found no association between current cannabis use or lifetime cannabis use at the time of survey completion and mortality.^17,18^ Collectively, almost no data are available on mortality risks associated with CUDs, and studies on the association between overall cannabis use and mortality is limited by using measures of cannabis use that are less clinically relevant and lacking biological plausibility (eg, lifetime ever use), inadequate adjustment for comorbid health factors, self-report for cannabis exposures, and lack of details on causes of death.

To address these gaps we completed a population-level study in Ontario, Canada’s most populous province (15.1 million residents in 2022), where medical cannabis has been widely available since 2015 and nonmedical cannabis was legalized in October 2018.^10^ We examined whether individuals with hospital-based (emergency department [ED] visit or hospitalization) care for CUD were at increased risk of all-cause mortality compared with the general population or individuals with hospital-based care for another substance. We examined differences in cause-specific mortality between individual hospital-based CUD care and the general population.

## Methods

### Study Design

We conducted a retrospective population-level cohort study of all individuals aged 15 to 105 years in Ontario, Canada. We included all individuals who were alive and eligible for the province’s public health insurance program, which provides universal access to all hospital and medically necessary physician-based services for 97% of residents of Ontario between January 2006 and December 2021, with follow-up until December 2022 for death. We identified all individuals with an incident CUD diagnosis and compared them with matched members of the general population or individuals with another substance use disorder diagnosis. This study was approved by the privacy office at ICES. ICES is a prescribed entity under Ontario’s Personal Health Information Protection Act (PHIPA). Section 45 of PHIPA authorizes ICES to collect and analyze personal health information without patient consent for approved research projects. The Strengthening the Reporting of Observational Studies in Epidemiology (STROBE) reporting guideline was followed in the reporting of this study.

### Data Sources

Clinical data capturing all ED visits, hospitalizations, and outpatient physician visits, along with sociodemographic characteristics, were obtained using 7 individual-level databases at ICES. Overall mortality was obtained from the Registered Persons Batabase (RPDB). Cause of death was obtained from the Office of the Registrar General Vital Statistics Database (ORGD), which records the cause of death from individual death certificates. These datasets were linked using unique encoded identifiers and analyzed at ICES (formerly the Institute for Clinical Evaluative Science). In Ontario, a coroner (who must be a licensed physician) must, by law, investigate all deaths from unnatural causes to determine the cause of death. Details on additional datasets are available in eMethods 1 in Supplement 1.

### Exposures

Hospital-based CUD care was defined as an ED visit or hospitalization with *International Statistical Classification of Diseases and Related Health Problems, Tenth Revision (ICD-10)* codes F12.X (mental and behavioral disorders due to use of cannabis) or T40.7 (poisoning by or adverse effects of cannabis, including derivatives) as the main or contributing reason for the visit. We used *ICD-9* and *Diagnostic and Statistical Manual of Mental Disorders* (Fifth Edition) codes 304.30 (cannabis dependence) and 305.20 (cannabis abuse) to identify hospitalizations in specialized mental health beds.

For a secondary analysis, we identified individuals with incident hospital-based care for other substance use disorders, including alcohol, opioids, or stimulants, and compared the risk of death between the substance use disorder types. We identified incident hospital-based care when an *ICD-10* or *DSM-5* code for the substance was the main or contributing reason for the visit (see eMethods 2 in Supplement 1 for codes by substance). All substance use disorder diagnoses were incident diagnoses defined as no prior hospital-based diagnoses for that substance in at least the past 3 years.

### Outcomes

The primary outcome was all-cause mortality. Secondary outcomes included cause-specific mortality using *ICD-10* diagnostic codes on death certificates using previously published methods.^19^ Each death could have more than 1 contributing cause, and we included immediate and underlying causes of death. We examined the following causes: alcohol poisoning, opioid poisoning, poisoning by other drugs, trauma (subdivided into motor vehicle collisions, fire, drowning, and falls and other accidental injuries), intentional self-harm, cancer (subdivided into lung cancer), infection, diseases of the circulatory system, respiratory system, and gastrointestinal system using previously established coding. See eMethods 3 in Supplement 1 for coding for different causes of death.^19^

### Covariates

We obtained sociodemographic information for each individual, including age, sex, rural residence, neighborhood income quintile, and whether or not they were immigrants to Canada since 1985. We obtained information on mental and substance use health care use in the 3 years before the index event, including outpatient mental health visits (primary care clinicians or psychiatrist) and ED visits and hospitalizations for substance use (alcohol, opioids, cocaine, amphetamines, and other) and mental disorders (mood, anxiety, self-harm, and other) using previously established coding.^20^ We identified previous diagnoses of 9 chronic health conditions using established coding.^21^ See eMethods 4 in Supplement 1 for covariate definition codes. Covariates in our study were complete except for rural residence and neighborhood income-quintile (0.60% missing).

### Statistical Analysis

#### Main Analysis

We used greedy matching in a 1:4 ratio to compare individuals with hospital-based CUD care to general population members. We matched on age, sex, and index date of the incident ED visit or hospitalizations for CUD. We compared the characteristics of individuals using descriptive statistics and standardized mean differences (SMD).^22^ Characteristics were obtained at the time of the incident hospital-based CUD care or assigned index date for matched comparators. We completed analyses on all-cause mortality until December 2022 and completed analyses on cause-specific mortality until December 2018. These time points reflect the end of data availability for overall mortality and cause of death.

For our primary analysis, we compared the risk of all-cause mortality between individuals with hospital-based CUD care to the matched general population using cumulative incidence functions and cause-specific Cox proportional hazard models. We adjusted for the following prespecified variables in 3 general categories. First, sociodemographics including age (in splines at the 5th, 27.5th, 50th, 72.5th, 95th percentiles), sex, neighborhood income quintile (6 levels including missing), immigrant status, and rurality (3 levels including missing). Second, comorbid mental health and substance use, including outpatient mental health care in the past 3 years (dichotomous family medicine, dichotomous psychiatry visit), substance hospital-based care (alcohol, cocaine, amphetamines, opioids, polysubstance use, other substance) in the past 3 years, and mental health hospital-based care (schizophrenia, depression, anxiety, deliberate self-harm in the past 3 years), and other mental health condition. Third, comorbid chronic conditions with treatment in the past 3 years, including hypertension, diabetes, asthma, cardiovascular disease, chronic obstructive pulmonary disease (COPD), cancer, kidney failure, and dementia. Given anticipated differences in mortality risk by age and sex, we conducted prespecified subgroup analyses examining risk and cause of death by age and sex strata.

For our secondary analysis comparing the risk of mortality for individuals with hospital-based care for different types of substance use disorders, individuals could experience up to 4 incident substance use disorders. We used robust sandwich covariance estimators to account for repeat measurements of unique individuals.

#### Sensitivity Analyses

We conducted 4 sensitivity analyses of our primary analysis. First, we included only individuals with no outpatient, or hospital-based care for mental or substance use disorders in the 3 years before their index date. Second, we used an unlimited lookback period for comorbid mental and substance use disorders and chronic health conditions. Third, we compared mortality risk when the cannabis code was the primary reason for the CUD visit compared with a contributing reason for the visit. Finally, we calculated an E-value estimating the hazard ratio [HR] required by an unmeasured confounder to explain away any observed association.^23^

Model statistical significance was determined by 95% CIs that did not cross 1. Statistical analyses were conducted using SAS Enterprise Guide 8.3 (SAS Institute) from September to December 2024.

## Results

During the study period, 11 622 571 individuals were eligible for inclusion in analysis, of whom 107 103 (0.9%) had incident hospital-based care for CUD (eFigure 1 in Supplement 1). Our primary matched analysis included 527 972 individuals (mean [SD] age, 29.9 [13.6] years; 330 034 [62.5%] male) with a median (IQR) follow-up of 5 (3-9) years, of which 106 994 had incident CUD. Individuals with hospital-based CUD care were more likely to live in low-income neighborhoods (29.0% vs 19.7% in the lowest income quintile) and be longstanding residents of Canada (91.1% vs 81.9%) compared with matched general population members. Individuals with hospital-based CUD care were more likely to have had an ED visit or hospitalization for substance use (38.9% vs 1.7%) or a mental disorder (34.8% vs 3.0%) in the past 3 years along with an outpatient mental health or addiction visit (68.2% vs 26.1%) relative to the general population. Individuals with hospital-based CUD care were more likely to have been diagnosed with several chronic conditions including hypertension (8.0% vs 6.6%), asthma (25.9% vs 19.7%), COPD (1.6% vs 0.4%), cardiovascular disease (1.1% vs 0.6%), and kidney failure (2.1% vs 0.6%) compared with the general population with no difference in prior cancer diagnoses (13.5% vs 13.1%) (Table 1). The annual number of cases of individuals with incident hospital-based CUD care increased by 6.1-fold during our study period (456 in 2006 to 3263 in 2021). See eFigure 2 in Supplement 1 for annual changes.

**Table 1.** Characteristics of Individuals With Incident Hospital-Based Care for Cannabis Use Disorder and the General Population

Characteristics	No. (%)		Standardized difference
	Cannabis use disorder (n = 106 994)	Matched general population (n = 420 978)	
Reason for cannabis use disorder visit^a^			
Intoxication	14 748 (13.8)	NA	NA
Harmful use	43 786 (40.9)	NA	NA
Dependence or withdrawal	9288 (8.7)	NA	NA
Cannabis-induced psychosis	5236 (4.9)	NA	NA
Amnesia, other, unspecified	5378 (5.0)	NA	NA
Cannabis poisoning	11 475 (10.7)	NA	NA
Mental health bed	18 996 (17.8)	NA	NA
Location of cannabis use disorder visit^b^			
Emergency department	78 746 (73.6)	NA	NA
Acute care hospital bed	9383 (8.8)	NA	NA
Specialized mental health hospital bed	20 750 (19.4)	NA	NA
Diagnostic code as main or contributing reason for visit			
Main	54 934 (51.3)	NA	NA
Contributing	52 060 (48.7)	NA	NA
Sex			
Male	67 023 (62.6)	263 011 (62.5)	0.003
Female	39 971 (37.4)	157 967 (37.5)	0.003
Age, mean (SD), y	29.79 (13.62)	29.88 (13.61)	0.006
15-18	21 276 (19.9)	82 648 (19.6)	0.006
19-24	29 882 (27.9)	117 252 (27.9)	0.002
25-44	38 805 (36.3)	153 462 (36.5)	0.004
45-64	14 629 (13.7)	58 183 (13.8)	0.004
≥65	2402 (2.2)	9433 (2.2)	0.000
Rurality			
Urban	94 116 (88.0)	377 708 (89.7)	0.06
Rural	12 246 (11.4)	41 533 (9.9)	0.05
Neighborhood income quintile			
1 (lowest)	31 000 (29.0)	82 758 (19.7)	0.22
2	22 531 (21.1)	82 337 (19.6)	0.04
3	19 193 (17.9)	83 649 (19.9)	0.05
4	17 284 (16.2)	84 427 (20.1)	0.10
5 (highest)	16 103 (15.1)	85 496 (20.3)	0.14
Long-term resident of Canada			
Yes	97 507 (91.1)	344 616 (81.9)	0.27
No	9487 (8.9)	76 362 (18.1)	0.27
Substance use acute care visits in past 3 y			
Any	41 591 (38.9)	7303 (1.7)	1.04
Alcohol	26 473 (24.7)	5749 (1.4)	0.74
Hallucinogens	1240 (1.2)	90 (0.0)	0.15
Cocaine	11 328 (10.6)	570 (0.1)	0.48
Amphetamines	4744 (4.4)	235 (0.1)	0.30
Opioids	5876 (5.5)	552 (0.1)	0.33
Polysubstance	12 064 (11.3)	1080 (0.3)	0.49
Other	3062 (2.9)	128 (0.0)	0.24
Mental health acute care visits in past 3 y			
Any	37 201 (34.8)	12 838 (3.0)	0.89
Mood disorder	17 049 (15.9)	4321 (1.0)	0.56
Anxiety disorder	18 958 (17.7)	7458 (1.8)	0.56
Deliberate self-harm	8287 (7.7)	1938 (0.5)	0.37
Other	6293 (5.9)	1645 (0.4)	0.32
Outpatient mental health and substance visits in past 3 y			
Any	73 013 (68.2)	109 922 (26.1)	0.93
Family physician	68 157 (63.7)	105 096 (25.0)	0.85
Psychiatrist	37 329 (34.9)	23 563 (5.6)	0.78
Any acute or outpatient mental health or substance visit in past 3 y			
Yes	84 151 (78.7)	114 287 (27.1)	1.20
No	22 843 (21.3)	306 691 (72.9)	0.93
Chronic health conditions in past 3 y			
Hypertension	8611 (8.0)	27 850 (6.6)	0.06
Asthma	27 697 (25.9)	82 857 (19.7)	0.15
Chronic obstructive pulmonary disease	1757 (1.6)	1883 (0.4)	0.12
Myocardial infarction or congestive heart failure	1190 (1.1)	2317 (0.6)	0.06
Dementia	345 (0.3)	526 (0.1)	0.04
Diabetes	5673 (5.3)	14 974 (3.6)	0.09
Cancer	14 460 (13.5)	55 030 (13.1)	0.01
Chronic kidney disease	2271 (2.1)	2681 (0.6)	0.13
Stroke	816 (0.8)	1553 (0.4)	0.05

Abbreviation: NA, not applicable.

^a^ Sums to more than 100% as individuals could have more than 1 cannabis code on presentation.

^b^ Sums to more than 100% as included individuals who presented to the emergency department (ED) and were admitted to hospital in both ED and hospitalizations.

Our sensitivity analysis of individuals without treatment for mental or substance use disorders in the past 3 years included 22 843 individuals (21.3%) with hospital-based CUD care and 306 691 matched members (72.9%) of the general population (Table 1). Our secondary analysis comparing individuals with hospital-based CUD care with other substance use disorders included 519 528 individuals, of which 372 820 (71.8%) had an incident hospital-based care for alcohol use disorder, 106 994 (20.6%) had an incident CUD, 78 985 (15.2%) had an incident stimulant use disorder, and 71 621 (13.8%) had an incident opioid use disorder. See eTable 1 and eFigures 3, 4, and 5 in Supplement 1 for cohort flows.

**Figure 1.**
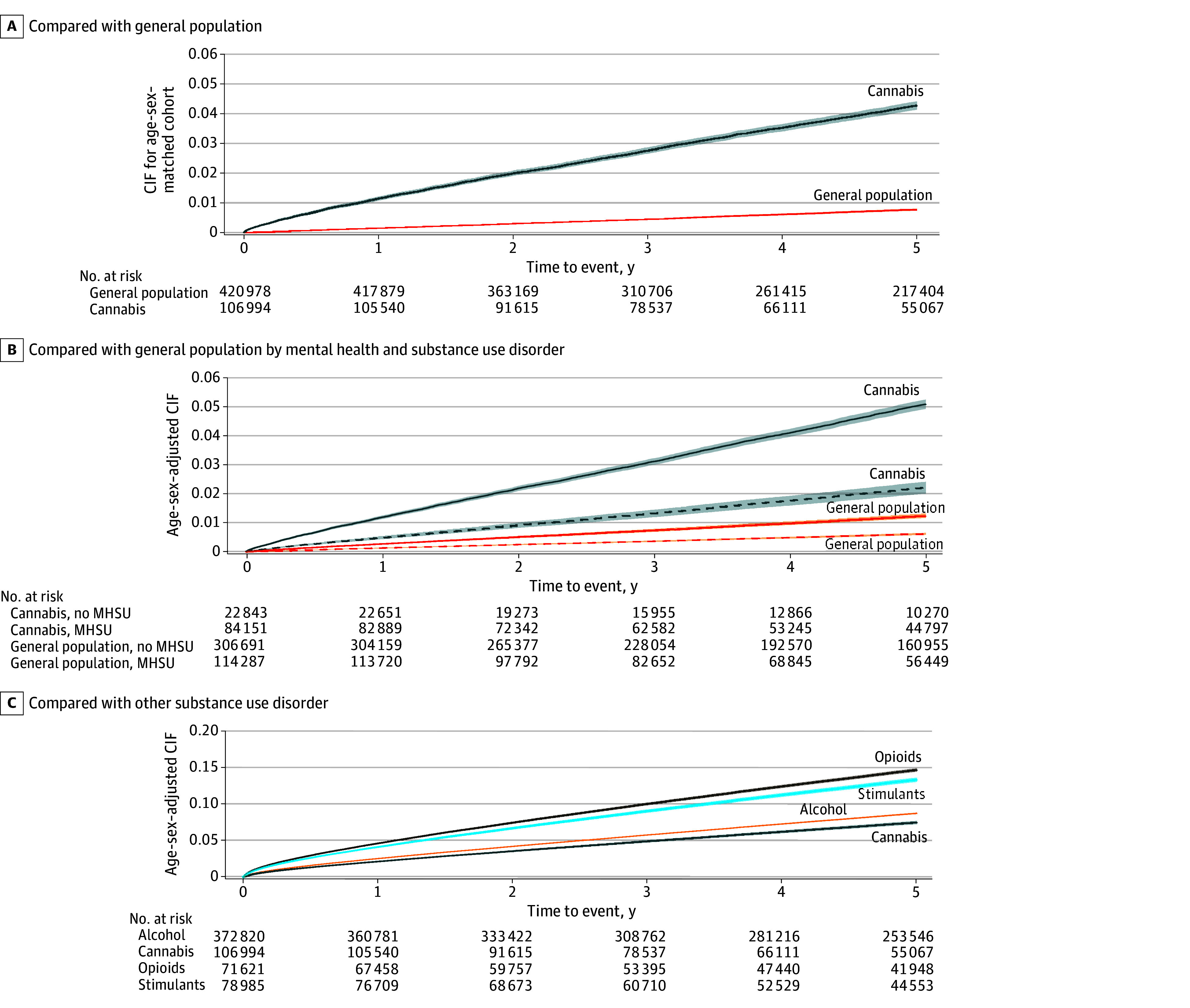
Cumulative Incidence Function (CIF) Curves Comparing the Risk of Death Over 5 Years A, The risk for individuals with hospital-based cannabis use disorder care and the matched general population. B, The risk for individuals with hospital-based cannabis use disorder care and the matched general population stratified by individuals with and without comorbid mental health and substance use (MHSU) disorders. Solid lines indicate individuals with a prior history of MHSU disorder and dashed lines indicate individuals without a prior history of MHSU disorder. C, The risk for hospital-based cannabis use disorder care vs other substance use disorders. Shaded regions represent 95% CIs.

Cumulative incidence functions for mortality risk over time for individuals with hospital-based CUD care and comparators are presented in Figure 1. Within 5 years of care for CUD, 3770 individuals (3.5%) died compared with 2550 members (0.6%) of the matched general population. After adjustment for sociodemographics, prior substance use, mental health care, and chronic conditions, the risk of death was greater (adjusted HR [aHR], 2.79 [95% CI, 2.62-2.97]; E-value, 5.0) for individuals with hospital-based CUD care relative to the general population. After excluding individuals with any outpatient, or hospital-based for mental health or substance use in the past 3 years, the absolute risk of death was diminished (5-year risk of death: 1.9% vs 0.5%), but the relative increase in risk was maintained for individuals with hospital-based CUD care relative to the general population (aHR, 2.60 [95% CI, 2.33-2.91]) (Table 2). Sensitivity analysis excluding individuals with any mental health or substance use care since the start of the study look-back in 2003 and comparing mortality risk for when the CUD diagnosis was the main or contributing reason for visit showed similar results (eTable 2 in Supplement 1).

**Table 2.** Risk of Mortality in Individuals With Hospital-Based Care for a Cannabis Use Disorder Compared With the General Population or Individuals With Hospital-Based Care for Other Substance Use Disorders

	No. at risk	Mortality, No. (%)				Life-years lost^b^	Crude rate^a^	HR (95% CI)^a^	
		Over maximum follow-up period available	1 y	5 y	10 y			Age- and sex-adjusted	Further adjusted^c^
**Primary analysis**									
Cannabis use disorder	106 994	6059 (5.7)	1216 (1.1)	3770 (3.5)	5299 (4.95)	1.8	892.7	5.96 (5.66-6.26)	2.79 (2.62-2.97)
General population	420 978	4458 (1.1)	627 (0.1)	2550 (0.6)	3804 (0.9)	0.2	152.6	1 [Reference]	1 [Reference]
**Sensitivity analysis, no hospital-based care for substance use disorders in the past 3 y**									
Cannabis use disorder	65 403	2315 (3.5)	454 (0.7)	1460 (2.2)	2048 (3.1)	0.6	575.3	3.96 (3.71-4.23)	2.54 (2.36-2.74)
General population	413 675	4055 (1.0)	556 (0.1)	2300 (0.6)	3450 (0.8)	0.2	140.1	1 [Reference]	1 [Reference]
**Sensitivity analysis, no hospital-based care for mental health or substance use disorders in the past 3 y**									
Cannabis use disorder	22 843	614 (2.7)	151 (0.7)	434 (1.9)	564 (2.5)	0.1	498.2	3.13 (2.81-3.49)	2.60 (2.33-2.91)
General population	306 691	2570 (0.8)	345 (0.1)	1466 (0.5)	2179 (0.7)	0.1	119.9	1 [Reference]	1 [Reference]
**Secondary analysis, cannabis use disorder compared with other substance use disorders**									
Cannabis use disorder	106 994	6059 (5.7)	1216 (1.1)	3770 (3.5)	5299 (5.0)	1.8	892.7	1 [Reference]	1 [Reference]
Alcohol use disorder	372 820	61 563 (16.5)	11 397 (3.1)	35 053 (9.4)	52 856 (14.2)	2.4	2198.6	1.11 (1.07-1.14)	1.30 (1.26-1.34)
Stimulant use disorder	78 985	10 004 (12.7)	2075 (2.6)	6108 (7.7)	8441 (10.7)	3.9	1908.4	1.92 (1.85-1.99)	1.69 (1.62-1.75)
Opioid use disorder	71 621	16 060 (22.4)	4027 (5.6)	10 623 (14.8)	14 486 (20.2)	4.9	3738.5	2.42 (2.33-2.51)	2.19 (2.10-2.27)

Abbreviation: HR, hazard ratio.

^a^ Mortality rates and hazard ratios at 5-year follow-up. Mortality rates per 100 000 person-years and hazard ratios at 5-year follow-up

^b^ Mean life-years lost per person for deaths occurring before 75 years of age.

^c^ Adjusted for age, sex, neighborhood income quintile, rurality, immigration status, past 3 years outpatient, emergency department, and hospital-based care for mental health (anxiety, depression, self-harm, psychosis, and other) and substance use disorders (alcohol, stimulants, opioids, other), previous diagnosis of chronic health conditions (hypertension, diabetes, asthma, cardiovascular disease, chronic obstructive pulmonary disease, cancer, kidney failure, dementia, and stroke). Hospital-based care was defined as an emergency department visit or hospitalization.

Our secondary analysis found that individuals with incident hospital-based CUD care were at a lower risk of death relative to individuals with another type of incident hospital-based substance use disorder. Alcohol use disorder (aHR, 1.30 [95% CI, 1.26-1.34]), stimulant use disorder (aHR, 1.69 [95% CI, 1.62-1.75]), and opioid use disorder (aHR, 2.19 [95% CI, 2.10-2.27]) were associated with increased risk of death at 5 years compared with individuals with hospital-based CUD care. See Figure 1C for cumulative incidence function and Table 2 for outcomes.

**Figure 2.**
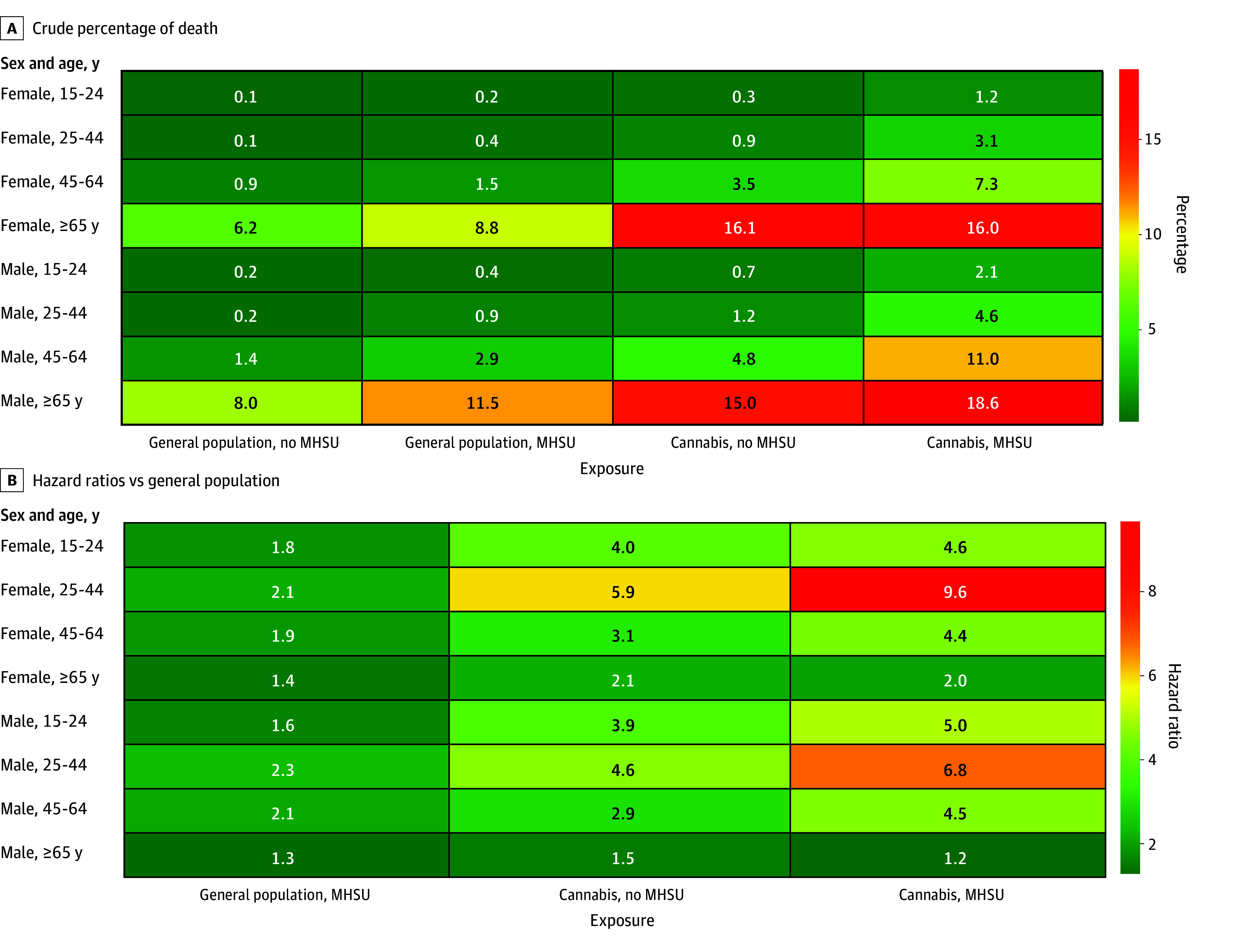
Proportion of Individuals With Hospital-Based Cannabis Use Disorder Who Died Within 5 Years and Hazard Ratios for Elevations in Risk The figure shows the crude percentage of death within 5 years (A) and the adjusted hazard ratios for individuals with hospital-based CUD care and matched members of the general population (B) stratified by individuals with and without a comorbid mental health or substance use disorder (MHSU). The reference group is the general population without a comorbid MHSU.

Figure 2 presents the proportion of individuals who died within 5 years based on age, sex, hospital-based CUD care, and comorbid substance and mental health disorders and HRs for elevations in risk. For both individuals with hospital-based CUD care and the general population, the absolute risk of death was higher in males and increased with age. Compared with the general population, there were larger relative increases in risk of death in younger individuals with hospital-based CUD care than in older individuals. For example, females aged 25 to 44 years with hospital-based CUD care and no comorbid mental health or substance use disorders were at a 5.9-fold increased risk of death relative to the general population (aHR, 5.91 [95% CI, 3.55-9.85]), compared with a 2.1-fold increased risk of death in women aged 65 years or older (aHR, 2.15 [95% CI, 1.62-2.84]) (eTable 3 in Supplement 1).

Table 3 presents the analyses of main and underlying causes of death at 5 years in individuals with hospital-based CUD care relative to the general population. Individuals with hospital-based CUD care were at increased risk of all investigated types of death, but at particularly elevated risk of death by suicide (aHR, 9.70 [95% CI, 6.04-15.57]), trauma (aHR, 4.55 [95% CI, 3.55-5.82]), opioid poisoning (aHR, 5.03 [95% CI, 2.86-8.84]), other drug poisonings (aHR, 4.56 [95% CI, 3.11-6.68]), and lung cancer (aHR, 3.81 [95% CI, 2.39-6.07]) relative to the general population. After excluding individuals with comorbid substance and health disorders, similar patterns of elevations in risk of death were observed (eTable 4 in Supplement 1). In individuals with hospital-based CUD care aged 15 to 44 years, a greater proportion of deaths involved substances, trauma, and self-harm (eTable 5 in Supplement 1); whereas in individuals aged 45 years and older, a greater proportion of deaths involved cancer and diseases of the circulatory and respiratory systems (eTable 6 in Supplement 1).

**Table 3.** Overall and Cause-Specific Risk of Mortality in Individuals With Hospital-Based Care for a Cannabis Use Disorder Compared With the General Population

Cause of death^a^	No. (%)		Rate per 100 000 person-years		Cause-specific death, HR (95% CI)	
	Matched general population (n = 268 506)	Cannabis use disorder (n = 68 862)	Matched general population	Cannabis use disorder	Crude	Adjusted
Total deaths	1576^b^	2390^b^	126.2	740.1	7.01 (6.48-7.59)	3.46 (3.13-3.82)
Substance-related	158 (10.0)	511 (21.4)	13.6	167.5	15.32 (12.10-19.39)	4.13 (3.07-5.55)
Alcohol poisoning	66 (4.2)	153 (6.4)	5.7	49.2	11.03 (7.61-15.98)	1.36 (0.82-2.27)
Opioid poisoning	39 (2.5)	168 (7.0)	3.6	54.8	20.49 (12.85-32.67)	5.03 (2.86-8.84)
Other drug poisoning	91 (5.8)	321 (13.4)	7.7	105.6	17.66 (12.91-24.14)	4.56 (3.11-6.68)
Trauma	248 (15.7)	400 (16.7)	22.1	129.1	7.68 (6.30-9.35)	4.55 (3.55-5.82)
Motor vehicle collision	96 (6.1)	84 (3.5)	8.5	26.6	3.88 (2.75-56.47)	2.26 (1.44-3.56)
Falls, drowning and fire	48 (3.0)	78 (3.3)	4.2	25.1	8.73 (5.50-13.86)	4.36 (2.43-7.82)
Other trauma	106 (6.7)	243 (10.2)	9.7	79.0	11.54 (8.59-15.50)	7.26 (5.09-10.36)
Intentional self-harm	36 (2.3)	165 (6.9)	3.0	55.4	19.80 (13.21-29.70)	9.70 (6.04-15.57)
Cancer	435 (27.6)	257 (10.8)	35.1	80.5	3.03 (2.52-3.65)	2.61 (2.08-3.28)
Lung cancer	82 (5.2)	75 (3.1)	6.6	24.2	5.05 (3.47-7.36)	3.81 (2.39-6.07)
Infection	51 (3.2)	61 (2.6)	4.2	18.9	4.83 (2.95-7.91)	2.65 (1.41-4.98)
Respiratory infection	65 (4.1)	71 (3.0)	5.3	22.3	4.51 (2.93-6.94)	1.42 (0.80-2.50)
Circulatory system diseases	342 (21.7)	361 (15.1)	28.7	115.2	4.29 (3.56-5.17)	2.09 (1.64-2.66)
Respiratory system diseases	148 (9.4)	189 (7.9)	12.2	59.15	5.71 (4.36-7.47)	2.36 (1.66-3.35)
Digestive system diseases	101 (6.4)	136 (5.7)	8.2	42.1	6.04 (4.32-8.45)	2.16 (1.36-3.42)
Other	180 (11.4)	384 (16.1)	15.7	125.1	10.89 (8.75-13.54)	4.86 (3.71-6.37)

Abbreviation: HR, hazard ratio.

^a^ Includes causes listed as the immediate and underlying causes on the death certificate and the percent sum to more than 100%.

^b^ This analysis only captured deaths until December 31, 2018, the date in which cause-specific death was available.

## Discussion

In this longitudinal population-based cohort study of 11.6 million people, we found that individuals with hospital-based care for CUD were at a 6-fold elevated risk of death compared with individuals of the same age and sex. There continued to be a 3-fold increased risk of death when trying to isolate the effect of CUD by accounting for further differences in sociodemographic and comorbid mental and substance use disorders and other chronic health conditions. Males and females with hospital-based CUD care of all ages were at elevated risk of death, but the relative increases in risk were the greatest in individuals aged 25 to 44 years. Individuals with hospital-based CUD care were at increased risk of all investigated types of death but at particularly elevated risk of death by suicide, trauma, opioid, alcohol, and other drug poisoning and lung cancer relative to the general population. Individuals with hospital-based CUD care had a slightly lower risk of death within 5 years than individuals with alcohol use disorder and about half the risk of death of individuals with hospital-based care for stimulants or opioids.

Despite large increases in the prevalence of CUDs, to date only a single study has examined whether individuals with CUDs are at increased risk of death. Adding to the literature, to our knowledge, we present the largest study to date on the longitudinal association between hospital-based treatment for a CUD and mortality. Our sample included 106 994 individuals with CUD care, of whom 6059 (5.7%) died by the end of follow-up, more than 43 times the next largest of prior population-based studies.^13,24^ CUDs may increase the risk of death through a variety of mechanisms. Exposure to and intoxication from THC has previously been associated with an increased risk of death by trauma, including motor vehicle collisions, violence, and death by suicide.^7,8,25^ The largest increases in cause-specific mortality in our study were for suicide (9.7-fold greater risk) and trauma (4.6-fold greater risk). THC has short-term hemodynamic effects and can increase blood pressure and heart rate and reduce cardiac perfusion, all of which may raise the risk of cardiovascular events and deaths.^26^ Long-term exposure to cannabis smoke and particulate matter might increase the risk of several chronic diseases, including cancer, chronic respiratory diseases and cerebrovascular disease.^27,28^ We observed more than double the risk of death from cardiovascular and respiratory disease and a 3.8-fold increase for lung cancer death in individuals with hospital-based CUD care than the general population. Individuals with CUDs have high rates of alcohol and tobacco use, and part of the elevated risk of death from the chronic disease may be driven by the harmful effects of tobacco and alcohol.^29,30,31^ However, emerging evidence suggests that cannabis use may be independently associated with cardiovascular disease.^28,32^ Deaths in individuals with CUD are also likely confounded by concurrent high-risk behaviors. In our study over 20% of deaths in individuals with hospital-based CUD care involved alcohol, opioids or another substance. Finally, CUD may also result in the development of severe mental health disorders, including schizophrenia and bipolar disease or worsen these disorders for individuals who already have them.^4,5^ Individuals with bipolar disorder and schizophrenia are at elevated risk of death compared with the general population.^33^ In addition, comorbid substance use disorders in individuals with bipolar disorder and schizophrenia have been shown to worsen treatment outcomes and increase the risk of death.^34^

We found that hospital-based CUD care was associated with slightly lower risk of death than hospital-based OUD care and a much lower risk than hospital-based StUD or OUD care However, frequent cannabis use and the use of higher-potency cannabis, the principal determinants of developing a CUD, are both increasing rapidly globally.^35,36^ In 2022, an estimated 17.7 million US individuals reported daily or near daily (DND) cannabis use, a higher number than those with DND drinking.^37^ Although our study cannot establish causality, individuals with a hospital-based diagnosis of CUD were at elevated risk of death. Although CUD may not be directly responsible, our findings highlight a growing segment of the population who are at elevated risk of death and may benefit from preventive measures.

### Limitations

Our study has limitations. First, not all individuals with CUD seek or access care, and our study did not have access to information on outpatient care for CUD. Consequently, our results capture a high-risk subpopulation of people with CUD and the findings may not generalize to CUDs that do not require hospital-based care. Second, we did not have detailed data on the length, frequency (eg, monthly vs daily) and type of cannabis used (smoked vs ingested) for individuals with a hospital-based CUD diagnosis in our study which may be relevant for the relationship between CUD and mortality. Similarly, there continue to be large gaps in the relationship between cannabis use in general (eg, not necessarily CUD) and mortality which were not evaluated in this study. Third, although we observed an association between hospital-based CUD care and mortality, unmeasured confounding (eg, tobacco use, risk-taking) may have biased our estimate away from the null. However, our E-value sensitivity analysis suggested that the observed HR of 2.8 for hospital-based CUD care and mortality could only be explained away by an unmeasured confounder that was associated with both CUD and mortality by an HR of 5.0 beyond measured confounders. This ratio is much greater than the reported association between smoking and all-cause mortality (risk ratio of 2.7 to 2.8 for current vs never smoker), making it unlikely that our findings are explained entirely by unmeasured confounding.^38,39^ In addition, sensitivity analyses excluding individuals with prior health care for mental or substance use disorders yielded similar associations to our primary analysis, highlighting elevated risk in populations without comorbid mental and substance use disorders.

## Conclusions

The results of this cohort study suggest that individuals who require hospital-based care for a CUD are at an elevated risk of premature death. Increases in mortality risk relative to the general population were greatest in individuals aged 25 to 44 years. Hospital-based CUD care was associated with a lower risk of mortality than individuals with care for other substances. However, large increases in regular cannabis use, and especially in forms known to predict CUD over time, highlight the importance of cannabis as a public health concern, especially in young strata of the population and in light of growing interest in cannabis legalization and market commercialization.

## Notes

Supplement 1. **eMethods 1.** Data Sources **eMethods 2.** Alcohol, Opioids, and Stimulant Exposure **eMethods 3.** All-Cause Mortality Codes for Outcome **eMethods 4.** Covariate Definitions **eFigure 1.** Cohort Flow for Cannabis Use Disorder and the General Population With Exclusions **eFigure 2.** Annual Count of Individuals With Hospital-Based CUD Care **eFigure 3.** Cohort Flow for Alcohol Use Disorder With Exclusions **eFigure 4.** Cohort Flow for Opioid Use Disorder With Exclusions **eFigure 5.** Cohort Flow for Stimulant Use Disorder and Exclusions **eTable 1.** Demographic Details of Individuals With Hospital-Based Care for Other Substance Use Disorders **eTable 2.** Sensitivity Analyses of 1) Primary Matched Analysis Excluding Individuals With Any Outpatient or Hospital-Based Mental Health or Substance Use Since 2003, 2) Secondary Analysis of Hospital-Based Care for Cannabis Use Disorders (CUDs) Compared to Other Substance Use Disorders Excluding Individuals With Co-Morbid Mental Health or Substance Use Care in Past 3 Years, and 3) Comparison of Mortality When the CUD Code is the Main or Contributing Reason for the Hospital-Based Care **eTable 3.** Five-Year Adjusted Hazard Ratios for All-Cause Mortality Stratified by Age, Sex, Hospital-Based Care for Cannabis Use Disorder (CUD) and Prior Care for a Mental Health or Substance Use Disorder (MHSU) **eTable 4.** Sensitivity Analysis for Overall and Cause-Specific Mortality Risk for Individuals With Hospital-Based Care for a Cannabis Use Disorder (CUD) and the General Population Excluding Individuals With Any MHSU in Past 3 Years **eTable 5.** Sensitivity Analysis for Overall and Cause-Specific Mortality Risk for Individuals With Hospital-Based Care for a Cannabis Use Disorder (CUD) and the General Population in Individuals Aged 15-44 **eTable 6.** Sensitivity Analysis for Overall and Cause-Specific Mortality Risk Individuals With Hospital-Based Care for a Cannabis Use Disorder (CUD) and the General Population for Individuals Aged 45+

Supplement 2.
Data Sharing Statement
