# Errors in Abstract, Results, and Table 1

**DOI:** 10.1001/jamanetworkopen.2025.5061

**Published:** 2025-03-17

In the Original Investigation titled “Cannabis Use Disorder Emergency Department Visits and Hospitalizations and 5-Year Mortality,”^1^ published February 6, 2025, there were errors in the Abstract, Results, and Table 1. The number and percentage of male individuals should have been listed as “330 034 (62.5%)” in both the Abstract and Results; the number and percentage of individuals in the matched general population who died within 5 years should have been listed as “2550 (0.6%)” in the Abstract; the percentage of the matched general population with diagnosed asthma should have been listed as “19.7%” in the Results; and in Table 1, for the category “Any acute or outpatient mental health or substance visit in past 3 y,” those with cannabis use disorder should have had “84 151 (78.7)” listed for Yes and “22 843 (21.3)” listed for No, and those in the matched general population should have had “114 287 (27.1)” listed for Yes and “306 691 (72.9)” listed for No. This article has been corrected.^1^
