# Supplementary material for: Cannabis Use Disorder Emergency Department Visits and Hospitalizations and 5-Year Mortality
Source: JAMA Netw Open. 2025 Feb 6;8(2):e2457852. doi: 10.1001/jamanetworkopen.2024.57852 (PMC11803479; doi:10.1001/jamanetworkopen.2024.57852)
Supplement: Supplement 1. — eMethods 1. Data Sources eMethods 2. Alcohol, Opioids, and Stimulant Exposure eMethods 3. All-Cause Mortality Codes for Outcome eMethods 4. Covariate Definitions eFigure 1. Cohort Flow for Cannabis Use Disorder and the General Population With Exclusions eFigure 2. Annual Count of Individuals With Hospital-Based CUD Care eFigure 3. Cohort Flow for Alcohol Use Disorder With Exclusions eFigure 4. Cohort Flow for Opioid Use Disorder With Exclusions eFigure 5. Cohort Flow for Stimulant Use Disorder and Exclusions eTable 1. Demographic Details of Individuals With Hospital-Based Care for Other Substance Use Disorders eTable 2. Sensitivity Analyses of 1) Primary Matched Analysis Excluding Individuals With Any Outpatient or Hospital-Based Mental Health or Substance Use Since 2003, 2) Secondary Analysis of Hospital-Based Care for Cannabis Use Disorders (CUDs) Compared to Other Substance Use Disorders Excluding Individuals With Co-Morbid Mental Health or Substance Use Care in Past 3 Years, and 3) Comparison of Mortality When the CUD Code is the Main or Contributing Reason for the Hospital-Based Care eTable 3. Five-Year Adjusted Hazard Ratios for All-Cause Mortality Stratified by Age, Sex, Hospital-Based Care for Cannabis Use Disorder (CUD) and Prior Care for a Mental Health or Substance Use Disorder (MHSU) eTable 4. Sensitivity Analysis for Overall and Cause-Specific Mortality Risk for Individuals With Hospital-Based Care for a Cannabis Use Disorder (CUD) and the General Population Excluding Individuals With Any MHSU in Past 3 Years eTable 5. Sensitivity Analysis for Overall and Cause-Specific Mortality Risk for Individuals With Hospital-Based Care for a Cannabis Use Disorder (CUD) and the General Population in Individuals Aged 15-44 eTable 6. Sensitivity Analysis for Overall and Cause-Specific Mortality Risk Individuals With Hospital-Based Care for a Cannabis Use Disorder (CUD) and the General Population for Individuals Aged 45+ [file jamanetwopen-e2457852-s001.pdf]

Supplemental Online Content

Myran DT, Pugliese M, McDonald AJ, et al. Cannabis use disorder ED visits and hospitalizations and 5-year mortality. *JAMA Netw Open.* 2025;8(2):e2457852. doi:10.1001/jamanetworkopen.2024.57852

eMethods 1. Data Sources

eMethods 2. Alcohol, Opioids, and Stimulant Exposure
eMethods 3. All-Cause Mortality Codes for Outcome
eMethods 4. Covariate Definitions
eFigure 1. Cohort Flow for Cannabis Use Disorder and the General Population With Exclusions
eFigure 2. Annual Count of Individuals With Hospital-Based CUD Care
eFigure 3. Cohort Flow for Alcohol Use Disorder With Exclusions
eFigure 4. Cohort Flow for Opioid Use Disorder With Exclusions
eFigure 5. Cohort Flow for Stimulant Use Disorder and Exclusions
eTable 1. Demographic Details of Individuals With Hospital-Based Care for Other Substance Use Disorders
eTable 2. Sensitivity Analyses of 1) Primary Matched Analysis Excluding Individuals With Any Outpatient or Hospital-Based Mental Health or Substance Use Since 2003, 2) Secondary Analysis of Hospital-Based Care for Cannabis Use Disorders (CUDs) Compared to Other Substance Use Disorders Excluding Individuals With Co-MorbidMental Health or Substance Use Care in Past 3 Years, and 3) Comparison of
Mortality When the CUD Code is the Main or Contributing Reason for the Hospital-Based Care
eTable 3. Five-Year Adjusted Hazard Ratios for All-Cause Mortality Stratified by Age, Sex, Hospital-Based Care for Cannabis Use Disorder (CUD) and Prior Care for a Mental Health or Substance Use Disorder (MHSU)
eTable 4. Sensitivity Analysis for Overall and Cause-Specific Mortality Risk for Individuals With Hospital-Based Care for a Cannabis Use Disorder (CUD) and the General Population Excluding Individuals With Any MHSU in Past 3-Years
eTable 5. Sensitivity Analysis for Overall and Cause-Specific Mortality Risk for Individuals With Hospital-Based Care for a Cannabis Use Disorder (CUD) and the General Population in Individuals Aged 15-44
eTable 6. Sensitivity Analysis for Overall and Cause-Specific Mortality Risk Individuals With Hospital-Based Care for a Cannabis Use Disorder (CUD) and the General Population for Individuals Aged 45+

This supplemental material has been provided by the authors to give readers additional information about their work.

© 2025 Myran DT et al. *JAMA Network Open*.

eMethods 1. Data Sources
We obtained study data from de-identified and linked health administrative databases housed at ICES. ICES is an independent, non-profit research institute funded by an annual grant from the Ontario Ministry of Health and Long-Term Care (MOHLTC). As a prescribed entity under Ontario's privacy legislation, ICES is authorized to collect and use health care data for the purposes of health system analysis, evaluation and decision support. Secure access to these data is governed by policies and procedures that are approved by the Information and Privacy Commissioner of Ontario. In 2018, the institute formerly known as the Institute for Clinical Evaluative Sciences formally adopted the initialism ICES as its official name.

The dataset from this study is held securely in coded form at ICES. While legal data sharing agreements between ICES and data providers (e.g., healthcare organizations and government) prohibit ICES from making the dataset publicly available, access may be granted to those who meet pre-specified criteria for confidential access, available at www.ices.on.ca/DAS (email: das@ices.on.ca). The full dataset creation plan and underlying analytic code are available from the authors upon request, understanding that the computer programs may rely upon coding templates or macros that are unique to ICES and are, therefore, either inaccessible or may require modification.

These datasets were linked using unique encoded identifiers and analyzed at ICES.
We used the following databases:

- National Ambulatory Care Reporting System (NACRS), which captures all ED visits and the cause of the visit within Ontario;
- Discharge Abstract Database (DAD), which includes records for all acute care hospitalizations in Ontario,
- Ontario Mental Health Reporting System Metadata (OMHRS) which includes all mental health hospitalizations in Ontario,
- OHIP Claims Database (OHIP, which captures all outpatient visits (including virtual) and the reason for visit in Ontario;
- Registered Persons Database (RPDB), which includes the total number of persons at-risk each month and individuals' age and sex; and
- Postal Code Conversation File+ (PCCF+) which contains information on the rurality (urban vs rural) and neighbourhood income for each person's home address.
- CIC-IRCC Permanent Residents database, which includes records for all landed immigrants, refugees, etc. in Ontario from January 1985 onwards
- Vital Statistics – Deaths (ORGD), which contains information on all deaths registered in Ontario including underlying causes of death and immediate cause of death information

© 2025 Myran DT et al. *JAMA Network Open*.

eMethods 2. Alcohol, Opioids, and Stimulant Exposure

We identified individuals with incident hospital-based care (an ED visit or hospitalization) due to substance use disorder when the *International Classification of Diseases (ICD) 10th revision* code was the main or contributing reason for the visit. Substances included alcohol use (ICD-10, F10X), opioid use (ICD-10, F11), and stimulant use (ICD-10, F15). This coding approach was taken are from the Canadian Institute for Health Information indicator "Hospital Stays for Harm Caused by Substance Use." (1)

© 2025 Myran DT et al. *JAMA Network Open*.

eMethods 3. All-Cause Mortality Codes for Outcome

Table. Definitions and data sources used for defining conditions at time of death

Conditions at Time of Death	ICD-10 Codes
Alcohol Poisoning	F10, T51.0, E244, G312, G621, G721, I426, K292, K700-K704, K709, K852, K860, R780, X45, Y15
Opioid Poisoning	T40.0; T40.1; T40.2; T40.3, F11
Poisoning by other drugs	T40.4, T40.5, T40.6, T40.7, T40.8, T40.9, T43, F12, F13, F14, F15, F16, F19
Motor Vehicle Collision	V01-V89, V91, V93-V99
Accidents (e.g., falls, drowning)	W00.-W19., V90, V92, W65-W74, X00-X19
Other Trauma	X85–Y09, W20-W64, W75-W99, X20-X39, X50, X59, Y20- Y36
Intentional Self Harm	X60–X84
Infection	A00-A99
Respiratory Infection	A15-A19, B97.4, J00-J22
Diseases of the circulatory system	I00 - I99
Diseases of the respiratory system	J00 - J99
Diseases of the digestive system	K00 - K95
Cancer	C00 - D49
Lung Cancer	C34.0, C34.1, C34.2, C34.3, C34.8, C34.9

© 2025 Myran DT et al. *JAMA Network Open*.

eMethods 4. Covariate Definitions

- Recent immigration status was determined using the Immigration Refugees and Citizenship Canada's Permanent Resident Database, which identifies all individuals who arrived in Ontario between 1985 and 2020.
- Urban or rural residence and neighbourhood-level income quintiles were defined using Statistics Canada census data. (2)
- Mental health ED visit or hospitalization in past 3 years and subtypes were defined using codes from Mental Health Codes from: Mental Health and Addictions Scorecard and Evaluation Framework indicator. (3)
- Substance use disorder ED visit or hospitalization in past 3 years and subtypes were defined using codes from Mental Health Codes from: Mental Health and Addictions Scorecard and Evaluation Framework indicator. (3)
- Outpatient substance use or mental health visits were defined using diagnostic and billing codes from Mental Health and Addictions Scorecard and Evaluation Framework indicator. (3)
- Chronic health conditions in past 3 years were defined using comorbidity indexes from previously validated ICD-10 coding algorithms. (4,5)

1. Ministry of Health and Long-Term Care. Early Psychosis Intervention Program Standards. Toronto, ON; 2011 Mar.
2. Statistics Canada. Population Centre and Rural Area Classification. 2016.
3. ICES. Mental Health and Addictions System Performance in Ontario: A Baseline Scorecard [Internet]. [cited 2024 Jun 2]. Available from: https://www.ices.on.ca/publications/research-reports/mental-health-and-addictions-system-performance-in-ontario-a-baseline-scorecard/
4. Van Walraven C, Austin PC, Jennings A, Quan H, Forster AJ. A modification of the Elixhauser comorbidity measures into a point system for hospital death using administrative data. Med Care [Internet]. 2009 Jun [cited 2024 Jun 2];47(6):626–33. Available from: https://pubmed.ncbi.nlm.nih.gov/19433995/
5. Quan H, Sundararajan V, Halfon P, Fong A, Burnand B, Luthi JC, et al. Coding algorithms for defining comorbidities in ICD-9-CM and ICD-10 administrative data. Med Care [Internet]. 2005 Nov [cited 2024 Jun 2];43(11):1130–9. Available from: https://pubmed.ncbi.nlm.nih.gov/16224307/

© 2025 Myran DT et al. *JAMA Network Open*.

eFigure 1. Cohort flow for cannabis use disorder and the general population with exclusions

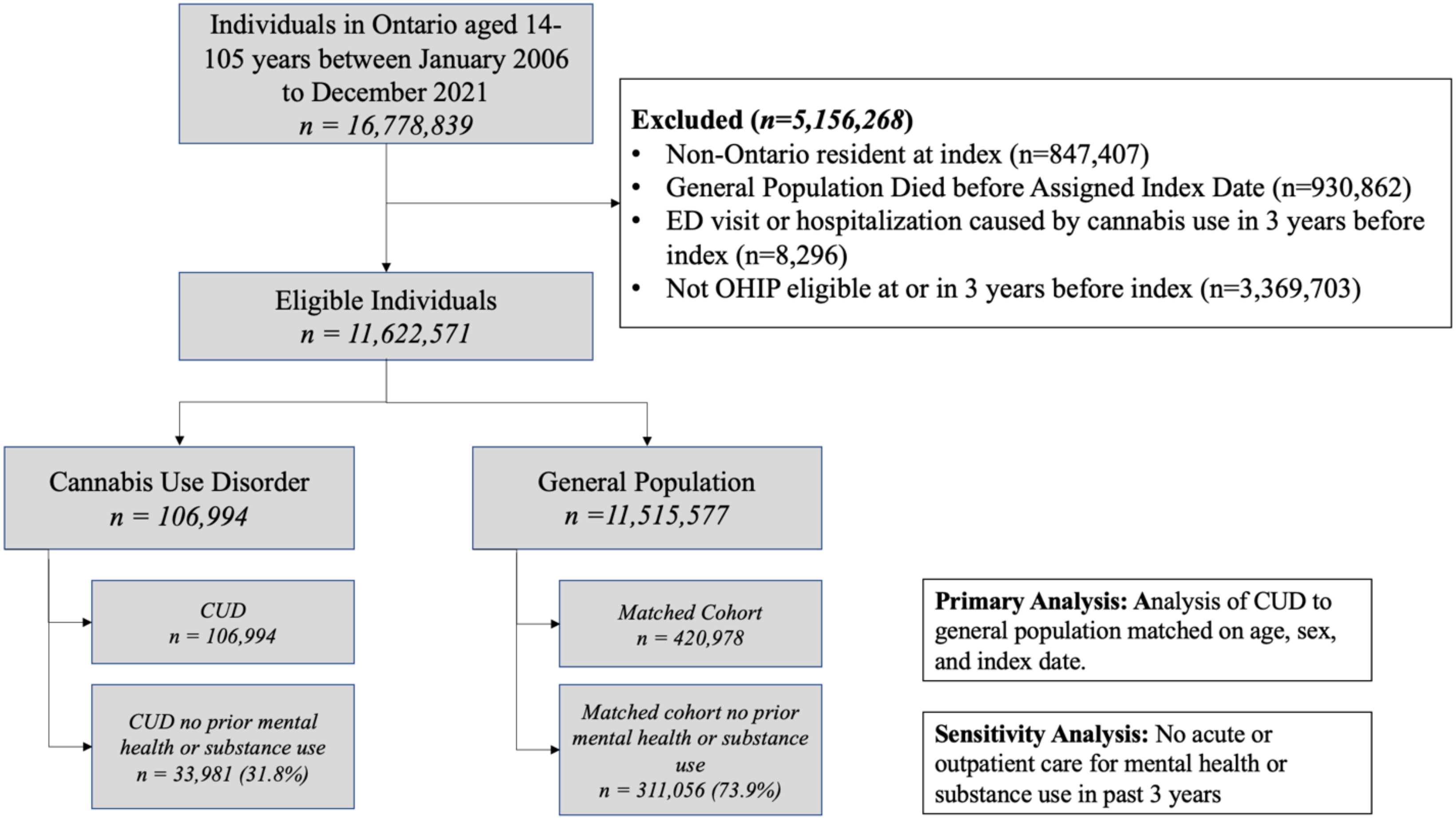

For 109 individuals with CUD there was no possible match in the general population based on age, sex and index date and these individuals were excluded from analysis.

© 2025 Myran DT et al. *JAMA Network Open*.

eFigure 2. Annual count of individuals with hospital-based CUD care. Legalization of non-medical cannabis occurred in October 2018 and access to medical cannabis was liberalized in 2014/2015.

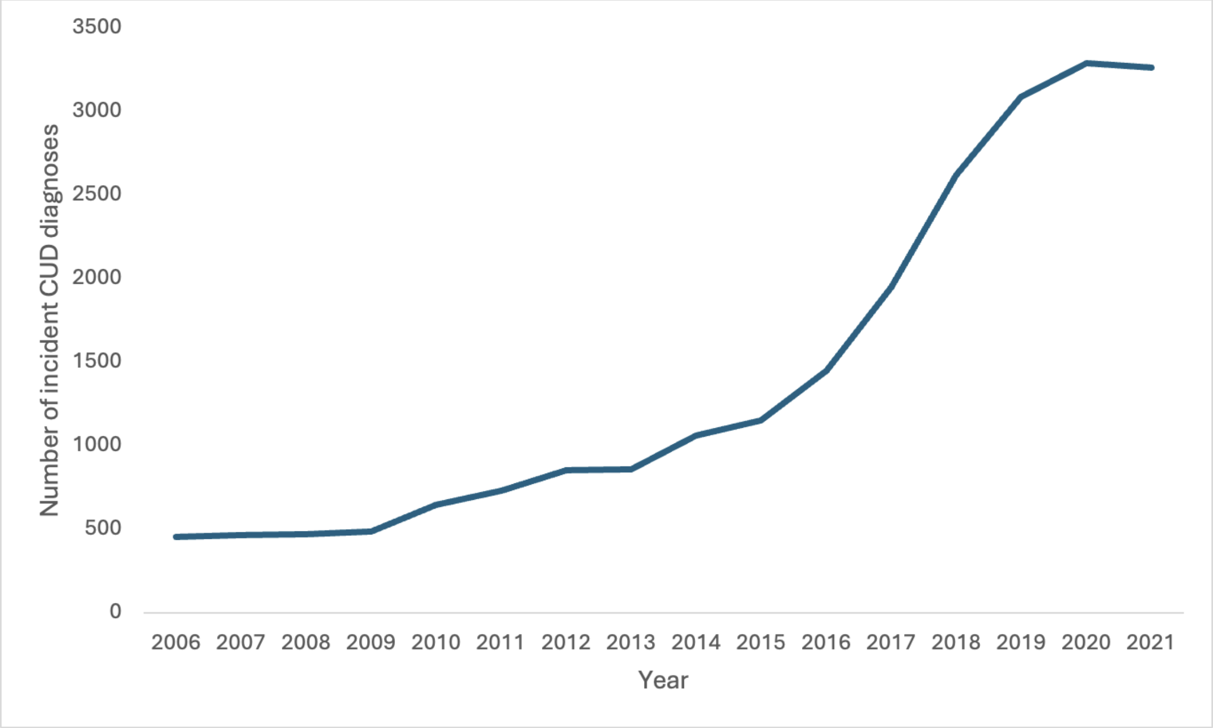

© 2025 Myran DT et al. *JAMA Network Open*.

eFigure 3. Cohort flow for alcohol use disorder with exclusions.

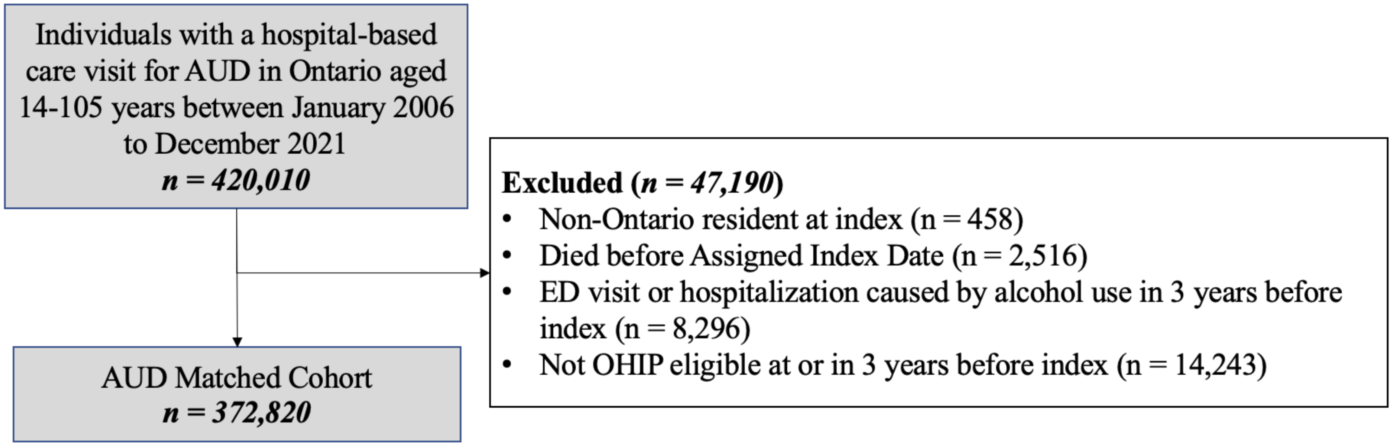

Abbreviations: AUD, Alcohol use disorder.

© 2025 Myran DT et al. *JAMA Network Open*.

eFigure 4. Cohort flow for opioid use disorder with exclusions.

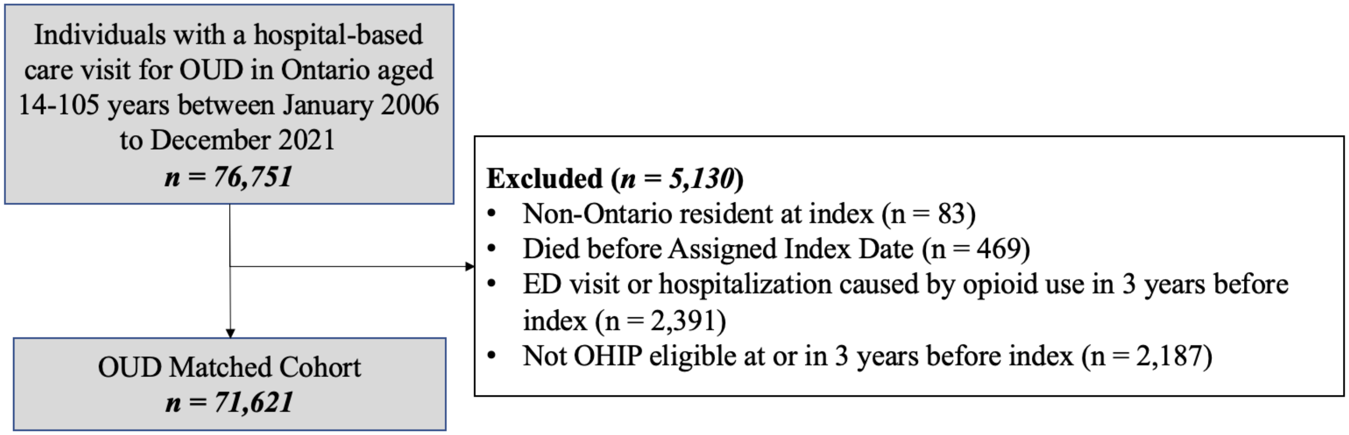

© 2025 Myran DT et al. *JAMA Network Open*.

eFigure 5. Cohort flow for stimulant use disorder and exclusions.

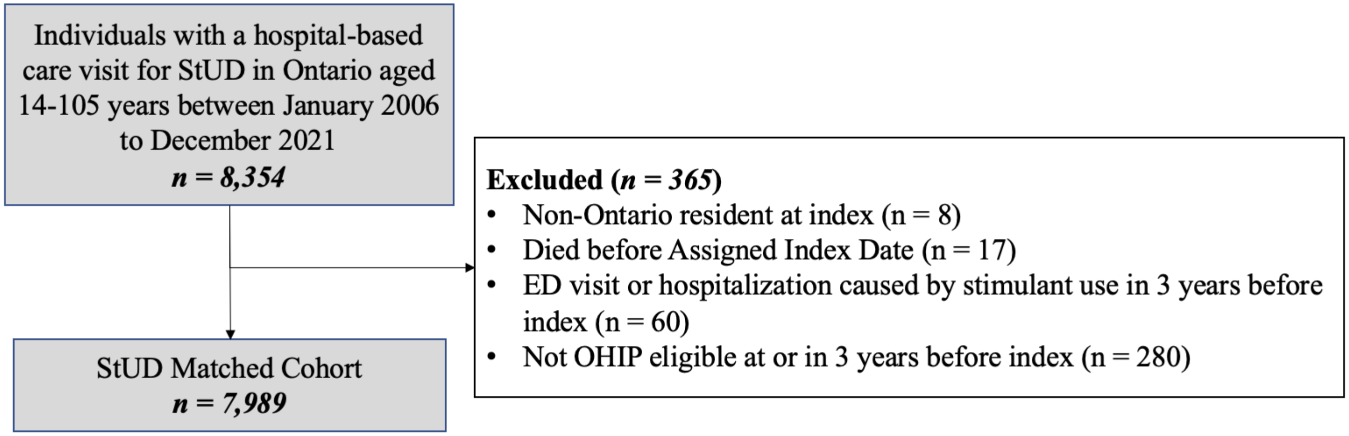

© 2025 Myran DT et al. *JAMA Network Open*.

eTable 1. Demographic details of individuals with hospital-based care for other substance use disorders.

	Alcohol (n=372,820)	Stimulants (n=78,985)	Opioids (n=71,621)
		N (%)	
**Sex**			
Male	137,307 (36.8)	26,591 (33.7)	29,871 (41.7)
Female	235,513 (63.2)	52,394 (66.3)	41,750 (58.3)
**Age**			
Mean ± SD	39.16 (19.01)	32.99 (11.82)	40.30 (16.76)
15-18 years	46,501 (12.5)	5,213 (6.6)	2,534 (3.5)
19-24 years	75,806 (20.3)	17,023 (21.6)	10,245 (14.3)
25-44 years	106,713 (28.6)	42,769 (54.1)	33,603 (46.9)
45-65years	99,154 (26.6)	13,122 (16.6)	18,321 (25.6)
65+ years	44,646 (12.0)	858 (1.1)	6,918 (9.7)
**Rurality**			
Urban	321,626 (86.3)	69,184 (87.6)	58,849 (82.2)
Rural	49,487 (13.3)	9,215 (11.7)	12,323 (17.2)
**Neighbourhood Income Quintile**			
1 (poorest)	99,635 (26.7)	26,918 (34.1)	25,223 (35.2)
2	76,544 (20.5)	16,998 (21.5)	15,221 (21.3)
3	67,901 (18.2)	13,218 (16.7)	11,972 (16.7)
4	62,973 (16.9)	11,155 (14.1)	9,872 (13.8)
5 (Richest)	62,040 (16.6)	9,770 (12.4)	8,473 (11.8)
**Long Term Resident of Canada**			
Yes	34,158 (9.2)	4,835 (6.1)	2,941 (4.1)
No	338,662 (90.8)	74,150 (93.9)	68,680 (95.9)
**Substance Use Acute Care Visits in Past 3 Years**			
Any	47,731 (12.8)	46,460 (58.8)	31,534 (44.0)
Alcohol	372,820 (100.0)	27,405 (34.7)	16,132 (22.5)
Hallucinogens	1,132 (0.3)	1,160 (1.5)	665 (0.9)
Cannabis	18,984 (5.1)	13,891 (17.6)	5,846 (8.2)
Cocaine	15,021 (4.0)	58,747 (74.4)	8,647 (12.1)
Amphetamines	3,864 (1.0)	22,545 (28.5)	4,460 (6.2)
Opioids	7,630 (2.0)	10,126 (12.8)	71,621 (100.0)
Polysubstance	16,944 (4.5)	16,712 (21.2)	14,268 (19.9)
Other	2,946 (0.8)	2,622 (3.3)	2,810 (3.9)
**Mental Health Acute Care Visits in Past 3 Years**			
Any	59,275 (15.9)	27,021 (34.2)	20,671 (28.9)
Mood Disorder	24,464 (6.6)	11,121 (14.1)	8,176 (11.4)

© 2025 Myran DT et al. *JAMA Network Open*.

Anxiety Disorder	30,560 (8.2)	14,263 (18.1)	10,924 (15.3)
Deliberate Self harm	15,174 (4.1)	8,304 (10.5)	7,402 (10.3)
Other	7,887 (2.1)	4,449 (5.6)	3,084 (4.3)
**Outpatient Mental Health and Addiction Visits in Past 3 Years**			
Any	195,271 (52.4)	59,097 (74.8)	56,967 (79.5)
Family Physician	186,033 (49.9)	56,138 (71.1)	55,114 (77.0)
Psychiatrist	65,300 (17.5)	28,183 (35.7)	23,361 (32.6)
**Any Acute or Outpatient MHA Visit in Past 3 Years**			
Yes	160,034 (42.9)	10,338 (13.1)	10,003 (14.0)
No	212,786 (57.1)	68,647 (86.9)	61,618 (86.0)
**Chronic Health Conditions in Past 3 Years**			
Hypertension	79,399 (21.3)	6,658 (8.4)	15,391 (21.5)
Asthma	74,486 (20.0)	19,509 (24.7)	18,128 (25.3)
Chronic Obstructive Pulmonary Disease	14,259 (3.8)	1,135 (1.4)	3,877 (5.4)
Myocardial Infarction or Congestive Heart Failure	18,207 (4.9)	1,892 (2.4)	5,372 (7.5)
Dementia	6,276 (1.7)	213 (0.3)	1,396 (1.9)
Diabetes	33,329 (8.9)	4,504 (5.7)	8,655 (12.1)
Cancer	67,470 (18.1)	9,640 (12.2)	15,026 (21.0)
Renal Failure	10,913 (2.9)	2,076 (2.6)	4,737 (6.6)
Stroke	7,500 (2.0)	666 (0.8)	1,642 (2.3)

© 2025 Myran DT et al. *JAMA Network Open*.

eTable 2. Sensitivity Analyses of 1) primary matched analysis excluding individuals with any outpatient or hospital-based mental health or substance use since 2003, 2) Secondary analysis of hospital-based care for cannabis use disorders (CUDs) compared to other substance use disorders excluding individuals with co-morbid mental health or substance use care in past 3 years, and 3) comparison of mortality when the CUD code is the main or contributing reason for the hospital-based care.

	No. at Risk	Mortality[A]	Mortality 1 Year	Mortality 5 Years	Mortality 10 Years	Life Years Lost[D]	Crude Rate[B]	Age and Sex Adjusted HR (95%CI)[B]	Further-adjusted HR (95%CI)[B,C]
**Sensitivity Analysis: Primary Matched Analysis excluding individuals with Mental health or Substance Use care since 2003**									
**Cannabis Use Disorder**	9,495	213 (2.2)	46 (0.5)	143 (1.5)	185 (2.0)	0.05	383.37	3.06 (2.55-3.67)	2.81 (2.34-3.37)
**Matched General Population**	205,042	1,589 (0.8)	170 (0.1)	778 (0.4)	1,261 (0.6)	0.09	92.58	Ref.	Ref.
**Sensitivity Analysis: Secondary Analysis CUD Compared to Other SUDs with no co-morbid mental health or substance use**									
**Cannabis Use Disorder**	22,843	614 (2.7)	151 (0.7)	434 (1.9)	564 (2.5)	0.65	498.23	Ref.	Ref.
**Alcohol Use Disorder**	160,034	26,869 (16.8)	5,414 (3.4)	15,807 (9.9)	23,381 (14.6)	1.83	2295.42	1.88 (1.71-2.07)	1.81 (1.65-1.99)
**Stimulant Use Disorder**	10,338	944 (9.1)	260 (2.5)	602 (5.8)	796 (7.7)	2.39	1409.15	2.78 (2.45-3.16)	2.55 (2.24-2.90)
**Opioid Use Disorder**	10,003	2,963 (29.6)	961 (9.6)	2,183 (21.8)	2,772 (27.7)	3.83	5672.66	3.98 (3.56-4.45)	3.54 (3.14-3.98)
**Sensitivity Analysis: Primary Analysis CUD code as the main or contributing reason for visit**									
**General Population**	420,978	4,458 (1.1)	627 (0.1)	2,550 (0.6)	3,804 (0.9)	0.2	152.6	Ref	Ref
**Cannabis Use Disorder Contributing Reason**	52,060	3,339 (6.4)	732 (1.4)	2,127 (4.1)	2,928 (5.6)	2.0	1050.1	7.64 (7.21-8.09)	3.19 (2.97-3.43)
**Cannabis Use Disorder Main Reason**	54,934	2,720 (5.0)	484 (0.9)	1,643 (3.0)	2,371 (4.3)	1.5	747.6	4.64 (4.34-4.94)	2.46 (2.29-2.64)

[A]Mortality over maximum follow up period available
[B]Mortality rates and hazard ratios at 5-years of follow up
[C]Adjusted for age, sex, neighbourhood income quintile, rurality, immigration status and previous diagnosis of chronic health conditions (hypertension, diabetes, asthma, cardiovascular disease, chronic obstructive lung disease, cancer, renal failure, dementia, and stroke).
[D]Average life years lost per person for deaths occuring before 75 years.

© 2025 Myran DT et al. *JAMA Network Open*.

eTable 3. Five-year adjusted hazard ratios for all-cause mortality stratified by age, sex, hospital-based care for cannabis use disorder (CUD) and prior care for a mental health or substance use disorder (MHSU)

Age and Sex	General population, no MHSU	General population, MHSU	CUD, no MHSU	CUD, MHSU
		Further-adjusted HR (95%CI)[A,B]		
**Female 15-24 y**	Ref.	1.80 (1.03-3.12)	4.03 (2.16-7.50)	4.55 (2.68-7.74)
**Female 25-44 y**	Ref.	2.11 (1.31-3.40)	5.91 (3.55-9.85)	9.61 (6.22-14.86)
**Female 45-64 y**	Ref.	1.86 (1.30-2.65)	3.13 (2.20-4.45)	4.42 (3.14-6.24)
**Female 65+ y**	Ref.	1.43 (0.93-2.20)	2.15 (1.62-2.84)	1.99 (1.25-3.16)
**Male 15-24 y**	Ref.	1.65 (1.22-2.23)	3.86 (2.87-5.20)	4.97 (3.81-6.49)
**Male 25-44 y**	Ref.	2.32 (1.80-2.99)	4.56 (3.40-6.12)	6.79 (5.37-8.57)
**Male 45-64 y**	Ref.	2.05 (1.66-2.54)	2.88 (2.28-3.64)	4.52 (3.69-5.52)
**Male 65+ y**	Ref.	1.29 (0.90-1.84)	1.55 (1.21-1.98)	1.23 (0.85-1.79)

[A]Mortality rates and hazard ratios at 5-years of follow up
[B]Adjusted for age, sex, neighbourhood income quintile, rurality, immigration status, past three years outpatient, ED, and hospital based care for mental health (anxiety, depression, self harm, psychosis and other) and substance us (alcohol, stimulants, opioids, other), previous diagnosis of chronic health conditions (hypertension, diabetes, asthma, cardiovascular disease, chronic obstructive lung disease, cancer, renal failure, dementia, and stroke).

© 2025 Myran DT et al. *JAMA Network Open*.

eTable 4. Sensitivity Analysis for overall and cause-specific mortality risk for individuals with hospital-based care for a cannabis use disorder (CUD) and the general population excluding individuals with any MHSU in past 3-years.

**Cause of Death**	**Matched General Population (n=198,409)**	**CUD (n=13,201)**	**Matched General Population (n=198,409)**	**CUD (n=13,201)**	**Age and Sex Adjusted HR (95%CI)[A]**	**Further-adjusted HR (95%CI)[A,B]**
	No. (%)		Rate per 100,000 Person Years			
**Total Deaths**	888 (0.4)	223	95.68	389.01	4.50 (3.77-5.36)	3.60 (3.00-4.31)
**Substance-related**	56 (6.3)	16 (7.2)	6.57	29.66	7.95 (3.99-15.85)	6.60 (3.25-13.39)
**Trauma**	140 (15.8)	29 (13.0)	17.35	57.57	4.32 (2.74-6.82)	4.17 (2.63-6.62)
**Intentional Self Harm**	19 (2.1)	15 (6.7)	2.15	26.17	11.98 (5.57-25.75)	11.59 (5.34-25.17)
**Cancer**	276 (31.1)	55 (24.7)	30.17	95.95	3.98 (2.86-5.55)	3.27 (2.33-4.59)
**Infection**	33 (3.7)	6 (2.7)	3.56	10.47	2.74 (1.00-7.49)	2.15 (0.77-5.98)
**Circulatory System Diseases**	204 (23.0)	45 (20.2)	23.16	81.99	3.30 (2.19-4.98)	2.32 (1.53-3.53)
**Respiratory System Diseases**	83 (9.3)	19 (8.5)	9.37	33.14	3.10 (1.72-5.58)	2.15 (1.18-3.90)
**Digestive System Diseases**	60 (6.8)	11 (4.9)	6.57	19.19	2.63 (1.07-6.47)	2.08 (2.86-5.55)

[A]Mortality rates and hazard ratios at 5-years of follow up

[B]Adjusted for age, sex, neighbourhood income quintile, rurality, immigration status and previous diagnosis of chronic health conditions (hypertension, diabetes, asthma, cardiovascular disease, chronic obstructive lung disease, cancer, renal failure, dementia, and stroke).

© 2025 Myran DT et al. *JAMA Network Open*.

eTable 5. Sensitivity Analysis for overall and cause-specific mortality risk for individuals with hospital-based care for a cannabis use disorder (CUD) and the general population in individuals aged 15-44

Cause of Death	Matched General Population (n=230,360)	CUD (n=59,204)	Matched General Population (n=230,360)	CUD (n=59,204)	Age and Sex Adjusted HR (95%CI)[A]	Further-adjusted HR (95%CI)[A,B]
	No. (%)		Rate per 100,000 Person Years			
**Total Deaths**	742 (0.3)	1407 (2.4)	68.08	494.27	8.57 (7.65-9.60)	7.51 (6.68-8.44)
**Substance-related**	121 (16.3)	383 (27.2)	11.93	142.98	15.62 (11.77-20.73)	13.83 (10.37-18.44)
**Trauma**	207 (27.9)	316 (22.5)	21.29	115.93	7.08 (5.70-8.80)	6.94 (5.56-8.65)
**Intentional Self Harm**	29 (3.9)	142 (10.1)	2.66	53.40	21.13 (13.41-33.29)	20.50 (12.93-32.51)
**Cancer**	124 (16.7)	52 (3.7)	11.56	18.27	1.84 (1.19-2.84)	1.76 (1.12-2.77)
**Infection**	15 (2.0)	22 (1.6)	1.84	7.73	5.21 (2.20-12.37)	2.17 (0.92-5.12)
**Circulatory System Diseases**	99 (13.3)	127 (9.0)	9.27	45.32	4.48 (3.14-6.39)	3.08 (2.12-4.49)
**Respiratory System Diseases**	45 (6.1)	54 (3.8)	4.40	18.97	4.42 (2.69-7.29)	2.48 (2.07-5.86)
**Digestive System Diseases**	34 (4.6)	47 (3.3)	3.12	16.51	4.93 (2.56-9.52)	3.32 (1.64-6.72)

[A]Mortality rates and hazard ratios at 5-years of follow up
[B]Adjusted for age, sex, neighbourhood income quintile, rurality, immigration status, past three years outpatient, ED, and hospital based care for mental health (anxiety, depression, self-harm, psychosis and other) and substance us (alcohol, stimulants, opioids, other), previous diagnosis of chronic health conditions (hypertension, diabetes, asthma, cardiovascular disease, chronic obstructive lung disease, cancer, renal failure, dementia, and stroke).

© 2025 Myran DT et al. *JAMA Network Open*.

eTable 6. Sensitivity Analysis for overall and cause-specific mortality risk individuals with hospital-based care for a cannabis use disorder (CUD) and the general population for individuals aged 45+

Cause of Death	Matched General Population (n=38,146)	CUD (n=9,658)	Matched General Population (n=38,146)	CUD (n=9,658)	Age and Sex Adjusted HR (95%CI)[A]	Further-adjusted HR (95%CI)[A,B]
	No. (%)		Rate per 100,000 Person Years			
**Total Deaths**	834 (2.2)	983 (10.2)	523.07	2568.47	5.72 (5.11-6.39)	3.88 (3.44-4.37)
**Substance-related**	37 (4.4)	128 (13.0)	25.09	350.13	14.67 (9.58-22.47)	10.35 (6.55-16.34)
**Trauma**	41 (4.9)	84 (8.5)	27.60	227.32	10.97 (6.80-17.70)	9.18 (5.58-15.10)
**Intentional Self Harm**	7 (0.8)	23 (2.3)	5.02	70.55	14.89 (6.04-36.72)	10.52 (4.10-27.02)
**Cancer**	311 (37.3)	205 (20.9)	196.31	543.48	3.46 (2.81-4.25)	2.58 (2.07-3.22)
**Infection**	36 (4.3)	39 (4.0)	23.21	101.90	4.63 (2.54-8.43)	3.06 (1.62-5.78)
**Circulatory System Diseases**	243 (29.1)	234 (23.8)	161.81	634.93	4.22 (2.29-5.26)	2.62 (2.07-3.31)
**Respiratory System Diseases**	103 (12.4)	135 (13.7)	65.85	357.97	6.33 (4.59-8.73)	3.15 (2.23-4.45)
**Digestive System Diseases**	67 (8.0)	89 (9.1)	42.65	232.55	6.49 (4.39-9.59)	4.95 (3.27-7.50)

[A]Mortality rates and hazard ratios at 5-years of follow up

[B]Adjusted for age, sex, neighbourhood income quintile, rurality, immigration status, past three years outpatient, ED, and hospital based care for mental health (anxiety, depression, self-harm, psychosis and other) and substance us (alcohol, stimulants, opioids, other), previous diagnosis of chronic health conditions (hypertension, diabetes, asthma, cardiovascular disease, chronic obstructive lung disease, cancer, renal failure, dementia, and stroke.

© 2025 Myran DT et al. *JAMA Network Open*.
