# Supplementary material for: Cannabis Use Disorder Emergency Department Visits and Hospitalizations and 5-Year Mortality
Source: JAMA Netw Open. 2025 Feb 6;8(2):e2457852. doi: 10.1001/jamanetworkopen.2024.57852 (PMC11803479; doi:10.1001/jamanetworkopen.2024.57852)
Supplement: Supplement 2. — Data Sharing Statement [file jamanetwopen-e2457852-s002.pdf]

Data Sharing Statement

Myran. Cannabis Use Disorder ED Visits and Hospitalizations and 5-Year Mortality. *JAMA Netw Open*. Published February 06, 2025. doi:10.1001/jamanetworkopen.2024.57852

Data
Data available: Upon request
How to access data: The data set from this study is held securely in coded form at ICES. Although data sharing agreements prohibit ICES from making the data set publicly available, access may be granted to those who meet pre-specified criteria for confidential access, available at https://www.ices.on.ca/useices-data/ The full data set creation plan and underlying analytic code are available from the authors on request, understanding that the computer programs may rely upon coding templates or macros unique to ICES and are therefore inaccessible or may require modification.

When available: With publication
